# Supplementary material for: A Thermophile-Fermented Compost Modulates Intestinal Cations and the Expression of a Juvenile Hormone-Binding Protein Gene in the Female Larvae of Hercules Beetle Dynastes hercules (Coleoptera: Scarabaeidae)
Source: Insects. 2023 Nov 27;14(12):910. doi: 10.3390/insects14120910 (PMC10744137; doi:10.3390/insects14120910)
Supplement: Supplementary file 1 [file insects-14-00910-s001.zip › insects-2712369-supplementary.pdf]

**Supplementary file**

Title:
A thermophile-fermented compost modulates intestinal cations and the expression of a juvenile hormone-binding protein gene in the female larvae of Hercules beetle *Dynastes hercules* (Coleoptera: scarabaeidae).

Authors:
Futo Asano, Taira Miyahara, Hirokuni Miyamoto and Hiroaki Kodama

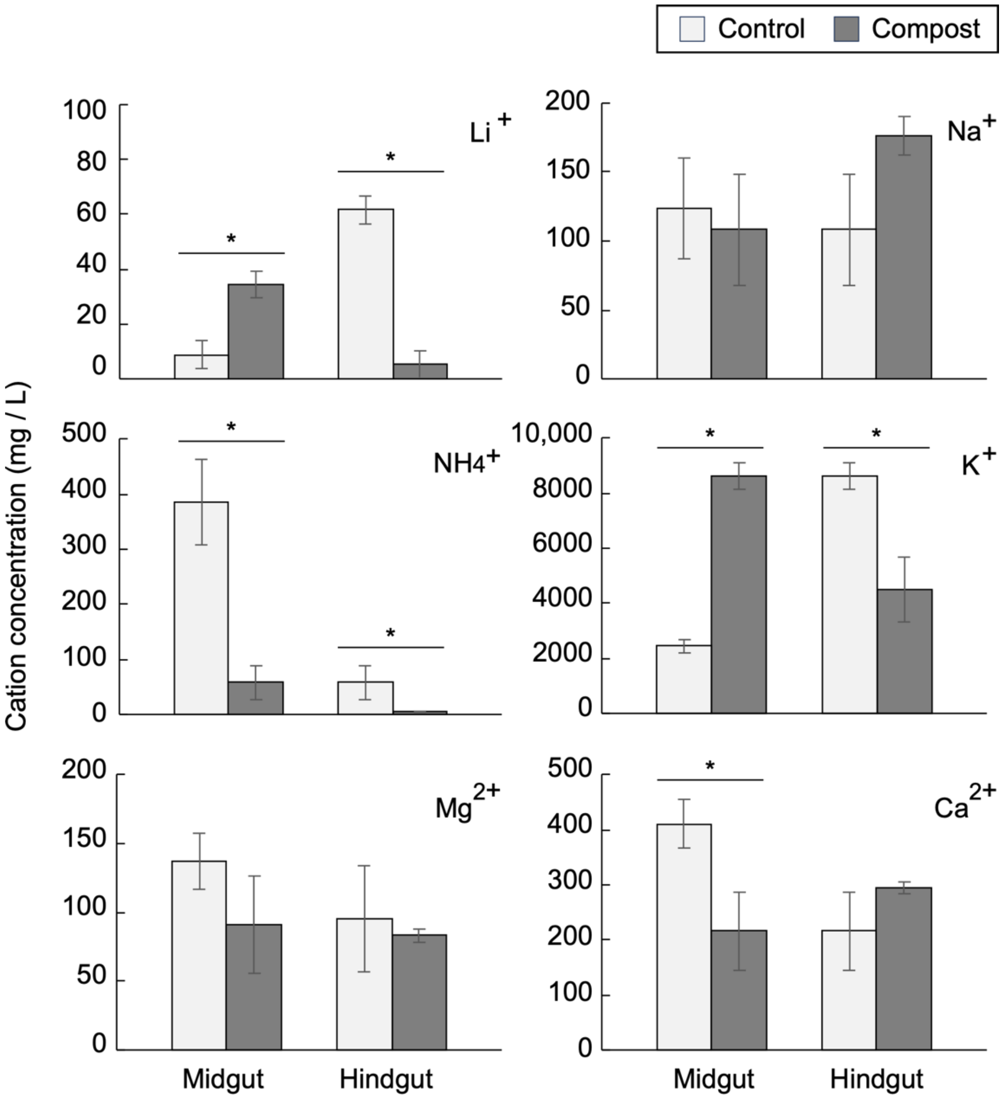


**Figure S1**. Cation concentrations in the intestinal fluids of male Hercules beetle larvae. Bars represent SD. $*p < 0.05$. $n = 4$.

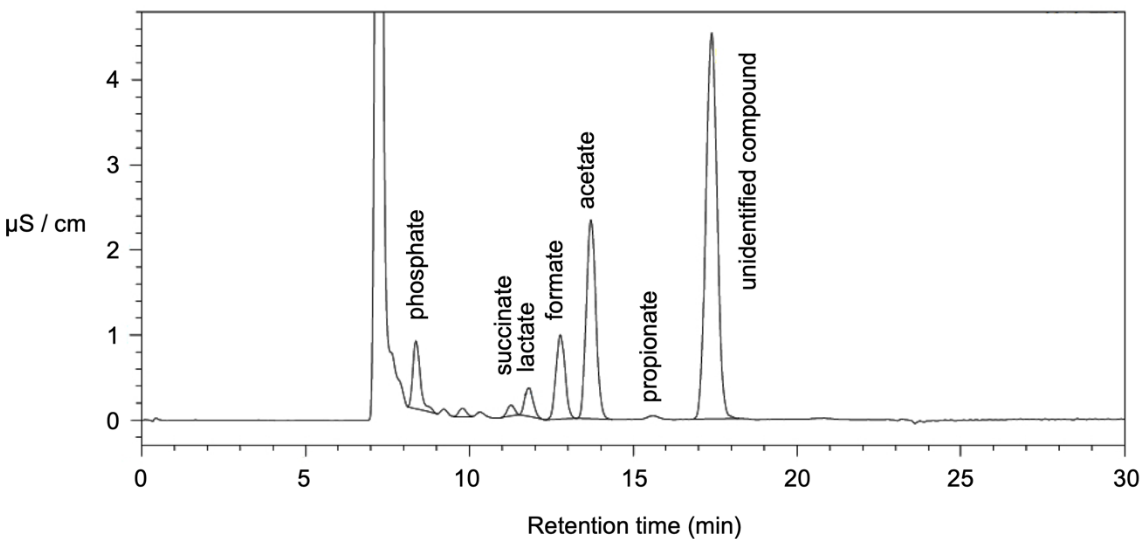


**Figure S2**. The chromatogram of HPLC. The midgut fluid of control female larvae was analyzed. Major acids are shown. A peak of approximately 17.5 min of retention time has not been identified. In this report, this unidentified peak is called RT-17.

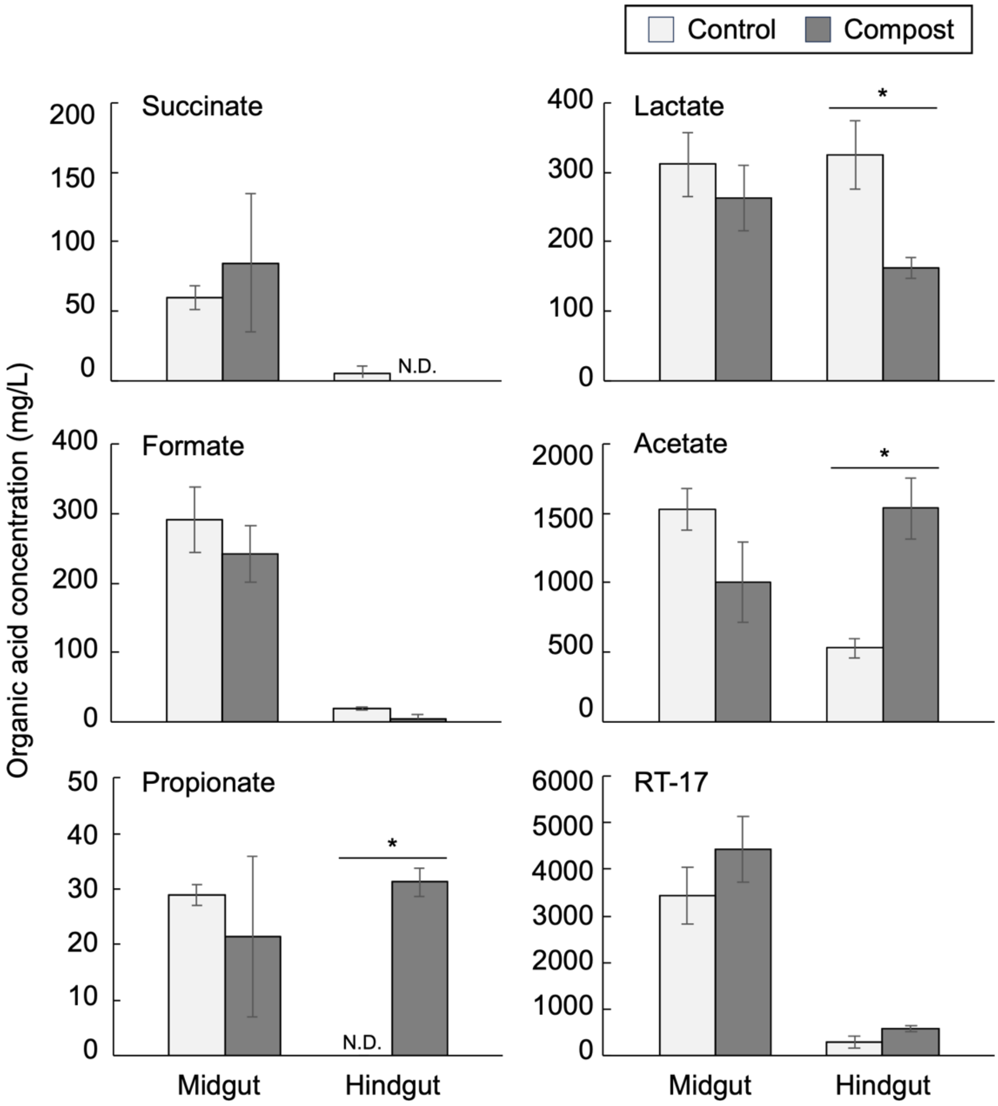


**Figure S3**. Organic acids in the male intestinal fluids. RT-17 is an unidentified compound with a retention time of approximately 17 min. N.D. represents not detected. Bars represent SD. *$p$ < 0.05. $n$ = 4.

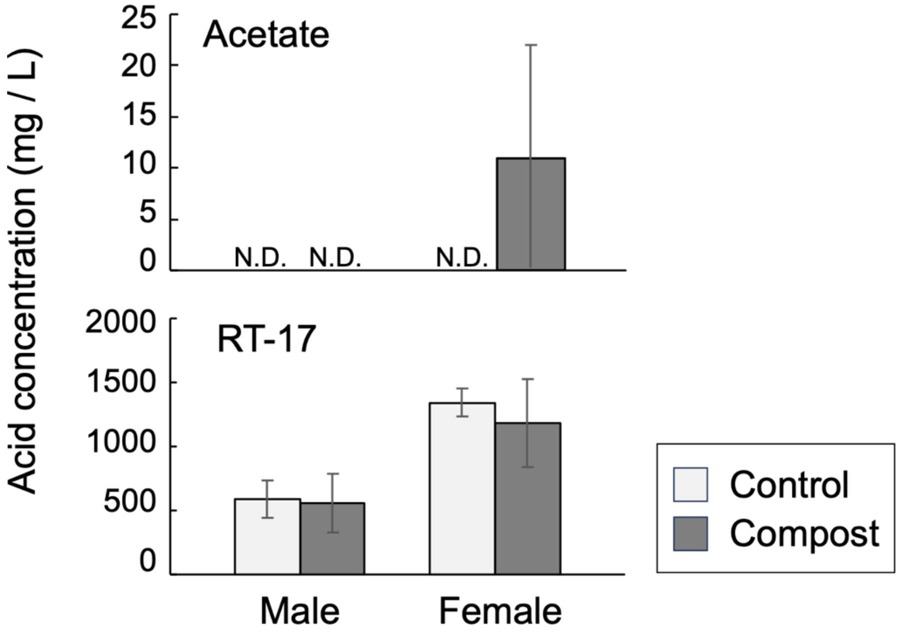


**Figure S4**. Concentrations of acetate and RT-17 in the feces of male and female Hercules beetle larvae. N.D. represents not detected. Bars represent SD. *n* = 4.

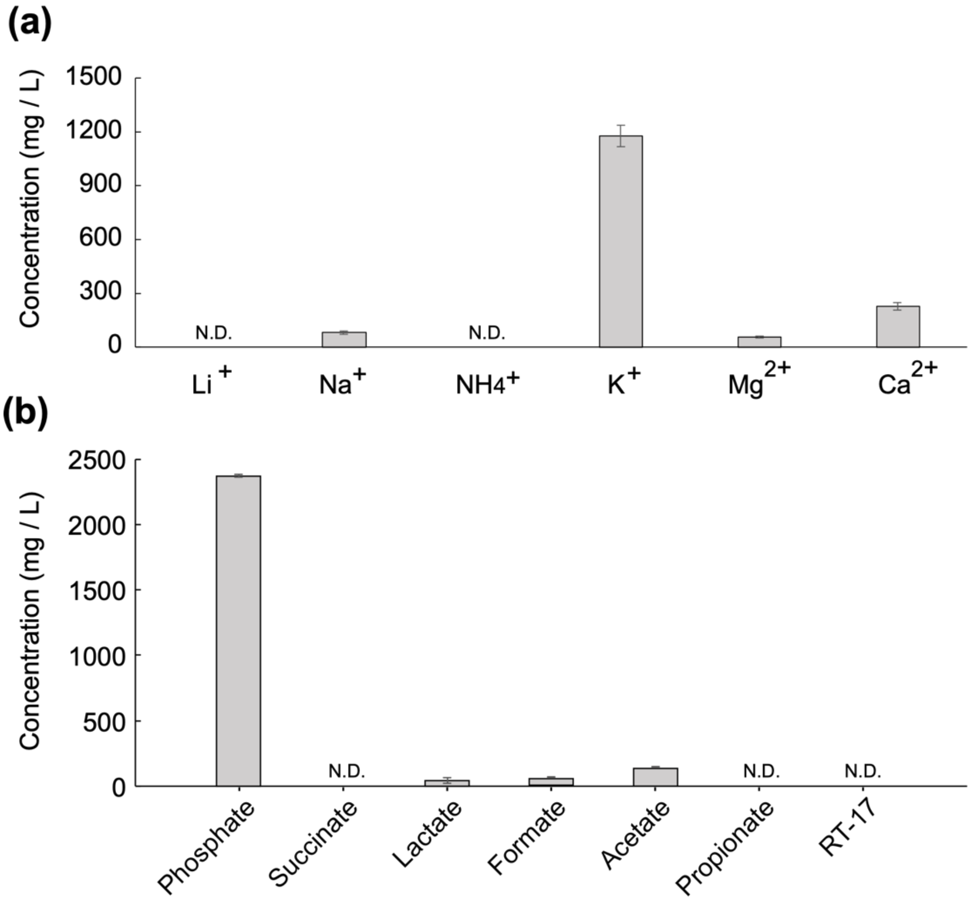


**Figure S5**. Cation, phosphate, and organic acid concentrations in humus. (**a**) Cation concentrations in the humus. (**b**) Phosphate and organic acid concentrations in the humus. N.D. represents not detected. Bars represent SD. *n* = 3.

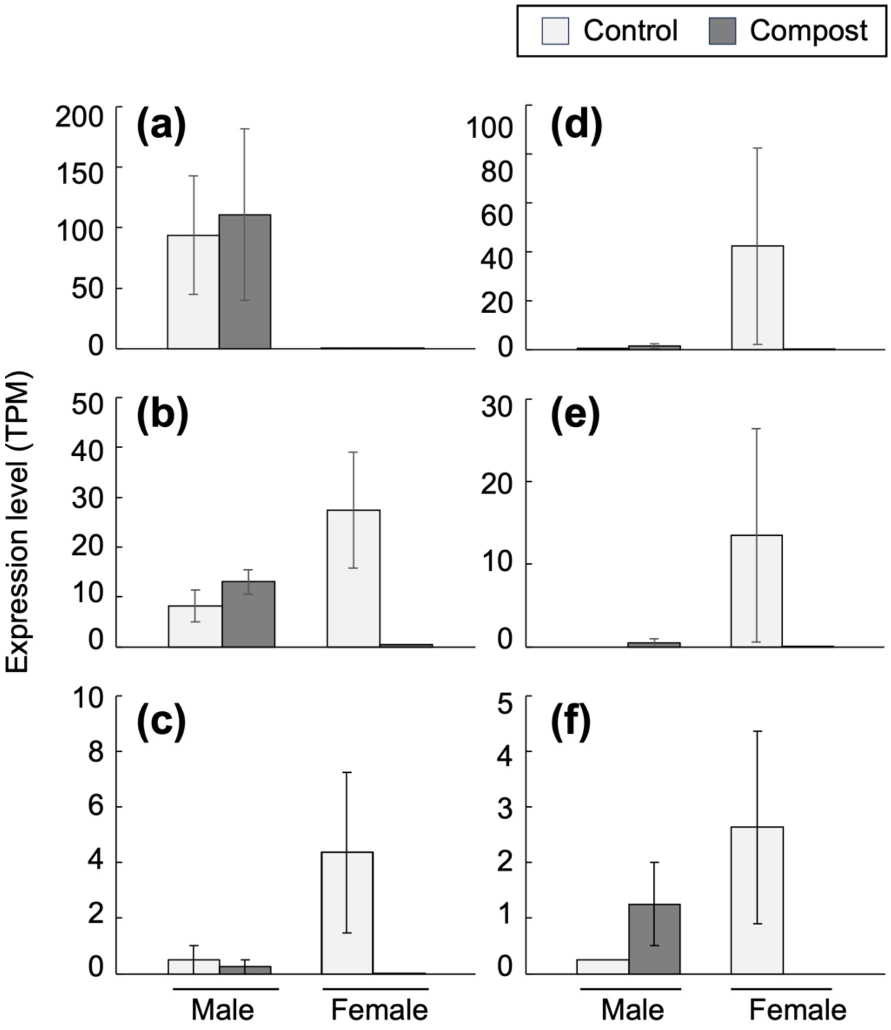


**Figure S6**. Expression patterns of six representative DEGs found in the comparison between female control larvae and compost larvae. These DEGs are listed as a category of peptidases/proteases (Table S2). The transcripts per million mapped reads (TPM) obtained from male and female larvae are collectively shown. This figure indicates the expression patterns of genes encoding the following proteins: (**a**) peptidase (DN1598_c1_g1_i20), (**b**) putative trypsin-like serine protease (DN2668_c0_g1_i1), (**c**) serine protease 6 (DN5342_c0_g2_i1), (**d**) serine protease 12 (DN27482_c0_g1_i1), (**e**) peptidase S1 domain-containing protein (DN28385_c0_g1_i1), and (**f**) serine protease (DN37931_c0_g1_i1). Bars represent SD. *n* = 4.

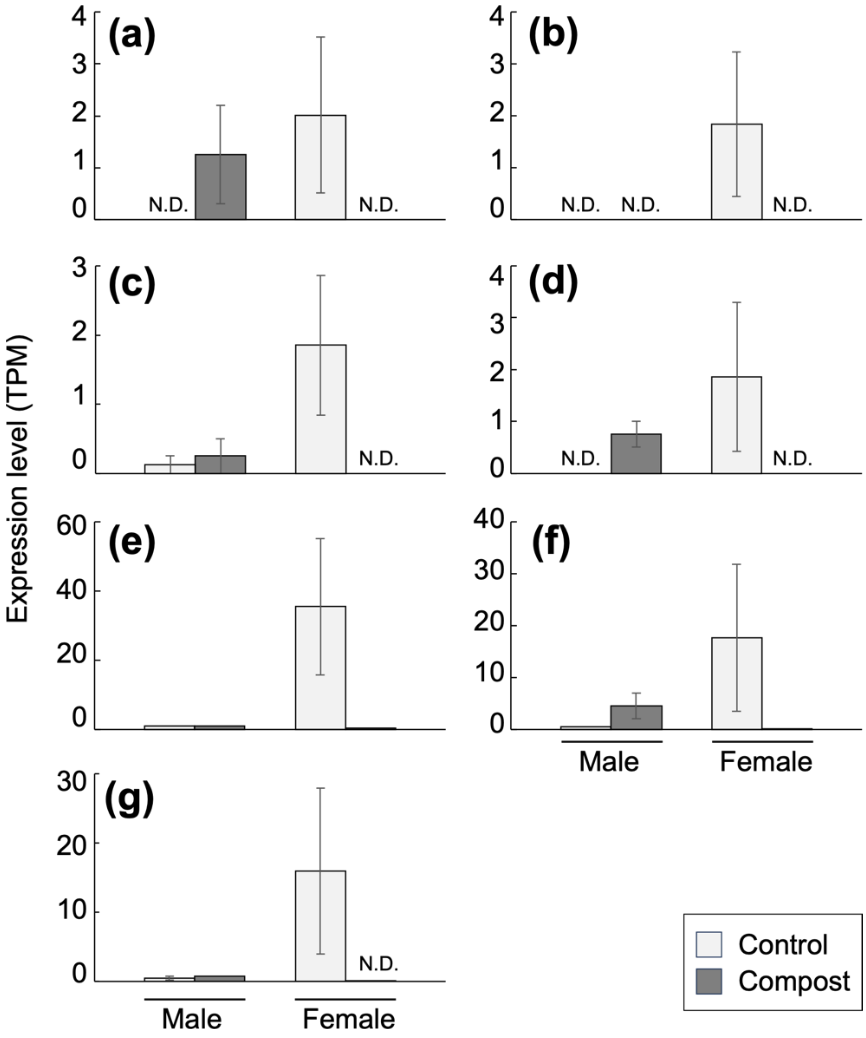


**Figure S7**. Expression patterns of seven representative DEGs found in comparison between the female control larvae and compost larvae. These DEGs are listed as a category for the peritrophic matrix-associated proteins (Table S3). The TPMs obtained from male and female larvae are collectively shown. This figure shows the expression patterns of genes encoding the following proteins; (**a**) putative peritrophic membrane matrix protein (DN961_c0_g1_i1), (**b**) putative peritrophic membrane matrix protein (DN961_c0_g1_i4), (**c**) chitin-binding type-2 domain-containing protein (DN2259_c0_g2_i3), (**d**) chitin-binding type-2 domain-containing protein (DN2259_c0_g3_i5), (**e**) chitin-binding type-2 domain-containing protein (DN5996_c0_g1_i1), and (**f**) putative chitin deacetylase (DN46633_c0_g1_i1), and (**g**) peritrophin (DN51506_c0_g1_i1). Bars represent SD. $n$ = 4.

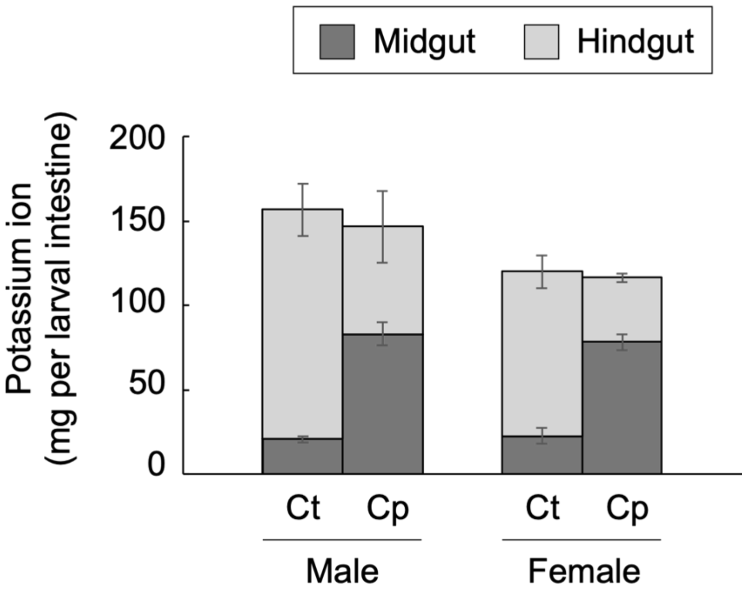


**Figure S8**. Intestinal amount of potassium ions in each larva. The total intestinal amount of potassium ions is further devided into the amount in the midgut and hindgut. Ct and Cp represent larvae from the control and compost groups, respectively. Bars represent SD. *n* = 4.

**Table S1**. (upregulated gene)

Table S1 List of the differentially expressed genes in a comparison of the two groups of female larvae with (Cp) and without (Ct) compost.

| Transcript_id | logFC (Cp/Ct) | logCPM | PValue | FDR | UniprotKB_accession | Description |
|---|---|---|---|---|---|---|
| DN1122_c1_g1_i4 | 14.26687827 | 6.887243058 | 1.24825E-11 | 9.41773E-08 | A0A0T6BCB0 | Hemolymph juvenile hormone-binding protein |
| DN1297_c0_g1_i5 | 10.91109704 | 3.554414577 | 4.21645E-06 | 0.003655186 | A0A482WDY8 | Frizzled and/or Fz domain containing protein |
| DN869_c0_g2_i12 | 10.23145812 | 2.886278925 | 2.30485E-18 | 6.95581E-14 | A0A8K0K5F9 | Uncharacterized protein |
| DN231_c0_g1_i4 | 10.15751696 | 2.813417536 | 1.37435E-08 | 4.14765E-05 | A0A8K0CAV3 | KASH domain-containing protein |
| DN5977_c0_g1_i9 | 10.07515229 | 2.7333425 | 2.49325E-08 | 6.27032E-05 | A0A0T6AUS4 | lysozyme |
| DN310_c0_g1_i22 | 9.862588129 | 2.527149098 | 7.17263E-08 | 0.000127331 | A0A5N4AK56 | Basement membrane-specific heparan sulfate proteoglycan core protein |
| DN6914_c0_g1_i1 | 9.749085559 | 2.447115473 | 3.51679E-16 | 5.30666E-12 | A0A0T6BAV3 | RNA binding protein (Fragment) |
| DN2157_c0_g1_i15 | 9.525930792 | 2.210811242 | 3.30479E-05 | 0.014885873 | A0A0T6B0V5 | Uncharacterized protein |
| DN7853_c0_g1_i3 | 9.287217895 | 1.9786336 | 2.60915E-07 | 0.000393708 | A0A0T6BCP6 | Centrosome-associated protein 350 |
| DN3378_c0_g1_i11 | 9.123039248 | 1.824386261 | 8.5774E-05 | 0.031567972 | A0A0T6AYD0 | Nuclear pore complex protein Nup98-Nup96 (Fragment) |
| DN4048_c0_g1_i6 | 9.109945547 | 1.806682338 | 2.92825E-11 | 1.76743E-07 | A0A0T6B9Q9 | RZZ complex subunit KNTC1/ROD C-terminal domain-containing protein |
| DN2157_c0_g1_i17 | 8.896277802 | 1.614391656 | 0.000120256 | 0.038199567 | A0A0T6B0V5 | Uncharacterized protein |
| DN878_c0_g1_i9 | 8.724378786 | 1.445507392 | 4.17593E-06 | 0.003655186 | A0A0T6BGD2 | Actin binding protein (Fragment) |
| DN2163_c0_g1_i10 | 8.724136813 | 1.444873473 | 2.11884E-10 | 9.13493E-07 | A0A0T6AWD9 | Ras-related protein Rab |
| DN4624_c0_g1_i4 | 8.638079247 | 1.365378351 | 1.36425E-09 | 4.57465E-06 | A0A0T6B1A7 | Zinc-finger associated domain containing protein |
| DN187_c0_g1_i8 | 8.511628877 | 1.244566829 | 7.41106E-05 | 0.028674146 | A0A1Y1MZA6 | Laminin subunit alpha (Fragment) |
| DN3713_c0_g1_i2 | 8.418356369 | 1.161516434 | 1.33628E-05 | 0.008129082 | A0A0T6AUJ6 | Armadillo repeat containing protein (Fragment) |
| DN1209_c0_g1_i47 | 8.301088666 | 1.056312917 | 6.72524E-08 | 0.000126997 | A0A0T6BD22 | Uncharacterized protein |
| DN4635_c0_g1_i1 | 8.274399575 | 1.052192397 | 2.76801E-05 | 0.013052483 | A0A0T6AZ15 | Protein kinase |
| DN333_c0_g1_i11 | 8.162771367 | 0.930701358 | 6.73303E-08 | 0.000126997 | A0A9N9SFU7 | Genome assembly, chromosome: 17 |
| DN2206_c0_g1_i53 | 8.114839625 | 0.885434077 | 2.17279E-07 | 0.00034512 | A0A1Y1NIG7 | Rho GTPase-activating protein 21 |
| DN419_c2_g1_i7 | 8.085380809 | 0.861620352 | 4.70178E-05 | 0.019985235 | A0A139WJA1 | RNA-directed DNA polymerase |
| DN2325_c2_g1_i11 | 7.979552621 | 0.863249066 | 6.01141E-07 | 0.000863897 | A0A0T6AWP7 | non-specific serine/threonine protein kinase |
| DN2186_c0_g1_i7 | 7.882763191 | 0.678288646 | 0.000121514 | 0.038199567 | A0A1Y1LA28 | PUM-HD domain-containing protein |
| DN36856_c0_g1_i2 | 7.846583973 | 0.645280103 | 8.91471E-05 | 0.032028229 | A0A0T6AVQ4 | ZZ-type domain-containing protein |
| DN1688_c1_g1_i4 | 7.843643444 | 0.647154378 | 0.000133418 | 0.039474783 | A0A0T6BGH3 | Zinc finger protein |
| DN6513_c0_g1_i23 | 7.710305107 | 0.528315256 | 1.47333E-06 | 0.001646802 | A0A0T6ATJ2 | Zinc-finger associated domain containing protein (Fragment) |
| DN56719_c0_g1_i2 | 7.686315103 | 0.509239114 | 1.87034E-06 | 0.001881502 | A0A0T6AWN7 | Histone deacetylase complex subunit SAP130 C-terminal domain-containing protein (Fragment) |
| DN3772_c0_g1_i1 | 7.630357458 | 0.464611901 | 0.000143087 | 0.041106412 | A0A9N9TD28 | Genome assembly, chromosome: 11 |
| DN4722_c0_g1_i17 | 7.490084218 | 0.342999354 | 6.81198E-06 | 0.004894733 | A0A8K0GIP2 | Protein kinase domain-containing protein |
| DN288_c0_g1_i9 | 7.435176181 | 0.29598826 | 6.35623E-06 | 0.004795618 | A0A0T6BH50 | Helicase |
| DN4736_c1_g1_i3 | 7.396992591 | 1.66006869 | 0.000119942 | 0.038199567 | A0A9N9SL10 | Genome assembly, chromosome: 4 |
| DN1314_c0_g1_i6 | 7.348690828 | 0.215408563 | 1.4741E-05 | 0.00839375 | A0A139WMV1 | Protein polybromo-1-like Protein |
| DN11435_c0_g1_i3 | 6.865026777 | 0.722031344 | 7.68907E-07 | 0.000988187 | A0A7F5RKE3 | Meiosis regulator and mRNA stability factor 1 |
| DN278_c0_g1_i6 | 6.798557294 | -0.230132446 | 4.65445E-05 | 0.019985235 | A0A0T6BBJ7 | C3H1-type domain-containing protein |
| DN3868_c0_g1_i4 | 6.783826524 | -0.246143858 | 9.77384E-05 | 0.03429821 | A0A0T6AZA8 | snRNA-activating protein complex subunit 3 |
| DN929_c0_g1_i2 | 6.694486552 | 1.762716338 | 3.10058E-05 | 0.014177618 | A0A8K0GJX2 | Rho GTPase-activating protein 190 |
| DN2522_c0_g1_i2 | 6.617238597 | -0.368708607 | 0.000104834 | 0.035951959 | A0A482VJC1 | Teneurin-m (Fragment) |
| DN1617_c0_g2_i1 | 6.564929081 | -0.409333018 | 0.000118184 | 0.038199567 | A0A0T6B4D3 | Protein phosphatase (Fragment) |
| DN2822_c0_g1_i8 | 6.564127152 | 0.304208544 | 1.83712E-05 | 0.010267137 | A0A8K0D098 | ABC transporter domain-containing protein |
| DN5658_c0_g2_i6 | 6.447262432 | -0.494230886 | 0.000131461 | 0.039474783 | A0A9N9SX76 | Genome assembly, chromosome: 2 |
| DN3515_c0_g1_i2 | 6.438616272 | -0.505065163 | 0.000173208 | 0.047520513 | A0A0T6BFV0 | ZZ-type domain-containing protein (Fragment) |
| DN2492_c0_g1_i3 | 6.426618609 | -0.507378351 | 0.000131514 | 0.039474783 | A0A1B6C1X0 | Reverse transcriptase domain-containing protein |
| DN1785_c0_g1_i1 | 6.415811013 | -0.517042075 | 0.000110413 | 0.037023992 | A0A8K0D415 | Rab-GAP TBC domain-containing protein |
| DN12354_c1_g1_i12 | 6.392770156 | -0.532111128 | 0.000137378 | 0.040251809 | A0A1Y1K217 | Lipid desaturase domain-containing protein |
| DN929_c0_g1_i32 | 6.119929232 | 1.688275524 | 0.00015771 | 0.044481605 | A0A8K0GJX2 | Rho GTPase-activating protein 190 |
| DN1851_c0_g1_i19 | 5.613874096 | 0.140358428 | 7.09299E-05 | 0.028541249 | A0A0T6B178 | Actin binding protein (Fragment) |
| DN3461_c0_g1_i45 | 5.55763963 | 0.270427167 | 7.34977E-05 | 0.028674146 | A0A8K0G470 | SAM domain-containing protein |
| DN534_c0_g1_i2 | 4.548406565 | 3.252113128 | 2.09205E-05 | 0.011274301 | A0A0T6AWW8 | Uncharacterized protein |

**Table S1**. continued. (downregulated gene)

| | | | | | | |
|---|---|---|---|---|---|---|
| DN12914_c0_g1_i2 | -1.993075406 | 7.26616014 | 2.85192E-05 | 0.013241258 | A0A4C1WQX8 | Uncharacterized protein |
| DN12439_c0_g1_i2 | -2.008923401 | 6.262000069 | 2.24601E-05 | 0.011891625 | A0A7M7MVX9 | Uncharacterized protein LOC113219380 |
| DN11698_c1_g3_i3 | -2.042368831 | 6.489206063 | 3.83398E-05 | 0.016768937 | A0A4C1WQX8 | Uncharacterized protein |
| DN11698_c0_g1_i1 | -2.080123224 | 4.231719832 | 3.77566E-05 | 0.016756723 | A0A7M7MVX9 | Uncharacterized protein LOC113219380 |
| DN7664_c0_g1_i3 | -2.082159754 | 5.909438642 | 7.27891E-05 | 0.028674146 | A0A9P0B9N2 | Genome assembly, chromosome: 5 |
| DN390_c0_g1_i7 | -2.09209741 | 9.226303409 | 1.27751E-05 | 0.008032051 | A0A8S9WK16 | Uncharacterized protein |
| DN12439_c0_g1_i3 | -2.142218869 | 7.31404677 | 1.34681E-05 | 0.008129082 | A0A8S9WK16 | Uncharacterized protein |
| DN390_c0_g1_i21 | -2.164583965 | 10.18997588 | 9.60761E-06 | 0.006443293 | A0A0M9A7Y2 | Uncharacterized protein |
| DN390_c0_g1_i12 | -2.197456456 | 6.969626292 | 7.98516E-06 | 0.00547691 | A0A5E4NGN7 | Uncharacterized protein |
| DN390_c0_g1_i14 | -2.229211248 | 7.893135754 | 3.94116E-06 | 0.003655186 | A0A151IRK4 | Uncharacterized protein |
| DN7664_c0_g1_i4 | -2.252835157 | 3.975470595 | 5.634E-05 | 0.023291589 | A0A9P0B9N2 | Genome assembly, chromosome: 5 |
| DN25909_c0_g1_i1 | -2.271507825 | 6.1916818 | 6.64086E-06 | 0.004888162 | A0A151IRK4 | Uncharacterized protein |
| DN11698_c1_g1_i1 | -2.297493514 | 7.882370443 | 4.23909E-06 | 0.003655186 | A0A8S9WK16 | Uncharacterized protein |
| DN11698_c1_g3_i2 | -2.317536374 | 7.716933123 | 1.6707E-06 | 0.001756993 | E0VHC5 | Uncharacterized protein |
| DN36512_c2_g1_i1 | -2.321762859 | 7.611963985 | 4.65881E-06 | 0.003799948 | A0A8S9WK16 | Uncharacterized protein |
| DN12439_c0_g1_i1 | -2.405123408 | 6.877966123 | 2.4985E-06 | 0.002432332 | A0A7M7MVX9 | Uncharacterized protein LOC113219380 |
| DN24812_c0_g1_i5 | -2.52313375 | 6.975643375 | 1.02033E-05 | 0.006694015 | #N/A | #N/A |
| DN1324_c0_g1_i5 | -2.617818291 | 3.588912799 | 7.95384E-05 | 0.030004852 | A0A6B9ESQ3 | Defensin (Fragment) |
| DN13229_c0_g1_i2 | -2.864212523 | 5.339811435 | 9.35484E-05 | 0.033214101 | A0A6P7G7J0 | Troponin I isoform X14 |
| DN920_c0_g1_i1 | -3.062504031 | 3.507273902 | 8.32987E-05 | 0.031035453 | #N/A | #N/A |
| DN30009_c0_g1_i1 | -3.402724707 | 2.935025081 | 0.000120255 | 0.038199567 | #N/A | #N/A |
| DN11160_c0_g1_i1 | -3.639477513 | 1.461598776 | 8.76565E-05 | 0.031872116 | A0A139WA62 | Uncharacterized protein |
| DN1598_c1_g1_i20 | -4.983063392 | 0.008422478 | 0.000100763 | 0.034953296 | A0A0T6B3L5 | Peptidase |
| DN2668_c0_g1_i1 | -5.967887546 | 4.12810758 | 3.40509E-12 | 3.4254E-08 | A0A5C0C9M4 | Putative trypsin-like serine protease |
| DN510_c1_g1_i9 | -5.9863649 | 2.633644159 | 0.000169964 | 0.047058286 | A0A0T6BH23 | Alpha-2-macroglobulin domain-containing protein |
| DN27989_c0_g1_i1 | -6.152470503 | 3.29244411 | 0.000131998 | 0.039474783 | A0A0T6B495 | Calcineurin-like phosphoesterase (Fragment) |
| DN3769_c0_g1_i3 | -6.42654098 | -0.514446947 | 0.000106655 | 0.03616557 | A0A0T6B1B6 | Cytochrome P450 (Fragment) |
| DN1979_c3_g1_i2 | -6.467469536 | -0.480793838 | 0.000133157 | 0.039474783 | #N/A | #N/A |
| DN7637_c0_g1_i2 | -6.559976109 | -0.409339032 | 0.000140739 | 0.040839891 | A0A0T6AZD1 | F-box domain-containing protein |
| DN2481_c0_g2_i1 | -6.603502258 | 1.047594849 | 7.55681E-05 | 0.028867956 | A0A5C0C9M9 | Uncharacterized protein |
| DN5996_c0_g1_i1 | -6.605703017 | 2.165017938 | 2.48966E-05 | 0.012317282 | A0A139WF85 | Chitin-binding type-2 domain-containing protein |
| DN1913_c0_g1_i4 | -6.659031818 | -0.334902183 | 0.000144381 | 0.041106412 | A0A1W4WBB5 | Protocadherin Fat 4 isoform X1 |
| DN5524_c0_g1_i9 | -6.804863929 | -0.227992615 | 5.76871E-05 | 0.023526219 | A0A8K0CTR5 | Pre-mRNA-splicing regulator female-lethal(2)D |
| DN5342_c0_g2_i1 | -6.94840072 | 1.351883686 | 1.09415E-05 | 0.007025583 | A0A8D4J1J6 | Serine protease 6 |
| DN13131_c0_g1_i3 | -7.293408432 | 1.571458343 | 1.00628E-06 | 0.001214742 | A0A0T6B9T9 | Large ribosomal subunit protein eL6 |
| DN2481_c0_g1_i1 | -7.340012934 | 3.491938364 | 6.21743E-11 | 3.12726E-07 | A0A5C0C9M9 | Uncharacterized protein |
| DN611_c1_g2_i7 | -7.583446265 | 0.409903542 | 1.40605E-05 | 0.008311387 | E2B455 | Uncharacterized protein (Fragment) |
| DN474_c0_g1_i6 | -7.677440031 | 0.497794089 | 4.48395E-06 | 0.003758917 | A0A8K0DAB3 | SAM domain-containing protein |
| DN46633_c0_g1_i1 | -7.682480225 | 3.952982206 | 1.31845E-07 | 0.000221053 | A0A5C0C9R1 | Putative chitin deacetylase |
| DN1851_c0_g1_i3 | -7.804559302 | 0.634882833 | 0.000118584 | 0.038199567 | A0A0T6B178 | Actin binding protein (Fragment) |
| DN37931_c0_g1_i1 | -7.912226079 | 0.709240935 | 5.32605E-05 | 0.022324306 | A0A5C0CAW9 | Serine protease |
| DN2259_c0_g2_i3 | -7.957117076 | 0.751698513 | 0.000167329 | 0.046757695 | A0A9J6BEU1 | Chitin-binding type-2 domain-containing protein |
| DN11432_c0_g1_i1 | -8.177198874 | 0.947766558 | 2.46594E-05 | 0.012317282 | A0A0T6AW22 | SET domain-containing protein |
| DN7853_c0_g1_i2 | -8.333751675 | 1.088968626 | 1.4321E-05 | 0.008311387 | A0A0T6BCP6 | Centrosome-associated protein 350 |
| DN2259_c0_g3_i5 | -8.395610366 | 1.145518649 | 2.71522E-05 | 0.013052483 | A0A9J6BEU1 | Chitin-binding type-2 domain-containing protein |
| DN51506_c0_g1_i1 | -8.401907549 | 2.557694467 | 5.74233E-08 | 0.000123784 | C0LL31 | Peritrophin |
| DN3793_c0_g1_i1 | -8.461179178 | 1.204173198 | 1.10175E-06 | 0.001278832 | A0A0T6B766 | RNA helicase |
| DN961_c0_g1_i4 | -8.482539036 | 1.223833344 | 0.000123504 | 0.038424903 | A0A5C0C9J0 | Putative peritrophic membrane matrix protein |
| DN1383_c1_g1_i1 | -8.627853337 | 1.353332384 | 6.28666E-06 | 0.004795618 | A0A0T6B2M1 | RNA binding protein (Fragment) |
| DN3013_c0_g1_i2 | -8.633660462 | 1.355892341 | 5.83006E-06 | 0.004630139 | A0A8K0GDK0 | Unkempt protein |
| DN3882_c0_g1_i11 | -8.712049129 | 1.429801833 | 1.69571E-08 | 4.65226E-05 | D6W8J3 | cysteine--tRNA ligase |
| DN28_c1_g1_i2 | -8.758408288 | 1.474302974 | 5.45273E-10 | 2.05697E-06 | D6WSR7 | Fukutin-related protein-like Protein |
| DN961_c0_g1_i1 | -8.851672589 | 1.56589305 | 2.46571E-05 | 0.012317282 | A0A5C0C9J0 | Putative peritrophic membrane matrix protein |
| DN28385_c0_g1_i1 | -9.083121423 | 3.19939181 | 7.1226E-07 | 0.000977059 | A0A8K0C9K5 | Peptidase S1 domain-containing protein |
| DN859_c0_g1_i24 | -9.153441626 | 1.847491567 | 1.68835E-06 | 0.001756993 | A0A0T6B271 | arginine--tRNA ligase |
| DN47266_c0_g1_i1 | -9.334224936 | 2.023110529 | 7.89027E-06 | 0.00547691 | A0A7M7G261 | MD-2-related lipid-recognition domain-containing protein |
| DN2897_c0_g1_i2 | -9.581384504 | 2.259085705 | 5.37996E-08 | 0.000123784 | A0A8K0GJC2 | Protein asunder |
| DN2502_c2_g1_i8 | -9.79719708 | 2.459188801 | 2.76093E-05 | 0.013052483 | A0A0T6B949 | Cytochrome P450 (Fragment) |
| DN1372_c0_g1_i3 | -9.804854652 | 2.474981877 | 2.32615E-05 | 0.012103608 | A0A8K0GHX5 | Myosin-IB |
| DN27482_c0_g1_i1 | -10.54270204 | 4.815969319 | 7.85861E-07 | 0.000988187 | B0ZBP0 | Serine protease 12 |
| DN299_c1_g1_i6 | -11.02559892 | 3.666068156 | 2.00921E-05 | 0.011024716 | A0A8A3SQ67 | NADH-ubiquinone oxidoreductase chain 5 |

**Table S2.**

Table S2 List of the six genes categorized as peptidases/proteases among the DEGs.

| Transcript_id | logFC | logCPM | PValue | FDR | UniprotKB_accession | Description |
|---|---|---|---|---|---|---|
| DN1598_c1_g1_i20 | -4.983063392 | 0.008422478 | 0.000100763 | 0.0349533 | A0A0T6B3L5 | Peptidase |
| DN2668_c0_g1_i1 | -5.967887546 | 4.12810758 | 3.41E-12 | 3.43E-08 | A0A5C0C9M4 | Putative trypsin-like serine protease |
| DN5342_c0_g2_i1 | -6.94840072 | 1.351883686 | 1.09E-05 | 0.00702558 | A0A8D4J1J6 | Serine protease 6 |
| DN37931_c0_g1_i1 | -7.912226079 | 0.709240935 | 5.33E-05 | 0.02232431 | A0A5C0CAW9 | Serine protease |
| DN28385_c0_g1_i1 | -9.083121423 | 3.19939181 | 7.12E-07 | 0.00097706 | A0A8K0C9K5 | Peptidase S1 domain-containing protein |
| DN27482_c0_g1_i1 | -10.54270204 | 4.815969319 | 7.86E-07 | 0.00098819 | B0ZBP0 | Serine protease 12 |

**Table S3.**

Table S3 List of the seven genes, including two isoforms categorized as proteins associated with the peritrophic matrix among DEGs.

| Transcript_id | logFC | logCPM | PValue | FDR | UniprotKB_accession | Description |
|---|---|---|---|---|---|---|
| DN5996_c0_g1_i1 | -6.605703017 | 2.165017938 | 2.49E-05 | 0.012317282 | A0A139WF85 | Chitin-binding type-2 domain-containing protein |
| DN46633_c0_g1_i1 | -7.682480225 | 3.952982206 | 1.32E-07 | 0.000221053 | A0A5C0C9R1 | Putative chitin deacetylase |
| DN2259_c0_g2_i3 | -7.957117076 | 0.751698513 | 0.000167329 | 0.046757695 | A0A9J6BEU1 | Chitin-binding type-2 domain-containing protein |
| DN2259_c0_g3_i5 | -8.395610366 | 1.145518649 | 2.72E-05 | 0.013052483 | A0A9J6BEU1 | Chitin-binding type-2 domain-containing protein |
| DN51506_c0_g1_i1 | -8.401907549 | 2.557694467 | 5.74E-08 | 0.000123784 | C0LL31 | Peritrophin |
| DN961_c0_g1_i4 | -8.482539036 | 1.223833344 | 0.000123504 | 0.038424903 | A0A5C0C9J0 | Putative peritrophic membrane matrix protein |
| DN961_c0_g1_i1 | -8.851672589 | 1.56589305 | 2.47E-05 | 0.012317282 | A0A5C0C9J0 | Putative peritrophic membrane matrix protein |
